# Study of chromatin diminution in *Cyclopskolensis* (Copepoda, Crustacea) by radiobiological methods

**DOI:** 10.3897/CompCytogen.v15.i4.64350

**Published:** 2021-09-28

**Authors:** Andrey Grishanin, Oksana Chinyakova

**Affiliations:** 1 Papanin Institute for Biology of Inland Waters Russian Academy of Sciences 152742, Borok Nekouzskii Raion, Yaroslavl Oblast, Russia Papanin Institute for Biology of Inland Waters Russian Academy of Sciences Borok Russia; 2 Dubna State University, 19, Universitetskaya str,141980, Dubna, Moscow Oblast, Russia Dubna State University Dubna Russia

**Keywords:** Copepoda, embryogenesis, radiation

## Abstract

The experimental results show that at doses of 20 Gy and 100 Gy, the development of *Cyclopskolensis* Lilljeborg, 1901 (Copepoda, Cyclopoida) embryos ceases at the 16-cell stage, without affecting the course of chromatin diminution. A dose of 200 Gy terminated the process of chromatin diminution in some of the embryos. These results support the hypothesis that cytoplasmic factors in the egg play an important role in the process of chromatin diminution.

## Introduction

The presence of chromatin diminution was carried out on the zooplanctonic crustacean *Cyclopskolensis* Lilljeborg, 1901 (Copepoda, Cyclopoida), in which the diminution process occurs at the stage of an 8-cell embryo (Grishanin et al. 1996). Chromatin diminution in Cyclopoida is the removal of a portion of the chromatin from chromosomes of the embryonic presomatic cells in one or two sequential cleavage divisions, while germ-line cells retain their nuclear DNA unchanged throughout ontogeny (Beermann 1977). The interphase of diminution divisions is significantly lengthened. Chromatin diminution in *C.kolensis* is a process of programmed removal of 90% of DNA from the nuclear genome of somatic line cells during the 4^th^ cleavage division, while the germ cell genome of these organisms remains unchanged (Zagoskin et al. 2010; Semeshin et al. 2011; Wyngaard et al. 2011). Chromatin diminution marks the timing of germline-soma differentiation: embryos prior to diminution possess a presomatic genome equal to that of the germline genome. After diminution the embyros possess what is referred to as a post somatic genome.

According to modern concepts of developmental biology, maternal genes of the egg determine the pattern of embryonic formation before fertilization and during initial cleavage divisions, after which the genes localized in the nuclei of embryonic cells play a role in the developmental process (Pomar and Jackle 1996; Jaeger 2018). The history of the study of factors controlling the chromatin diminution goes back to the beginning of the 20^th^ century, when the discoverer of chromatin diminution, Theodore Boveri, conducted an experiment to determine these factors. Boveri (1910) showed in *Ascaris* (Nematoda) Karl Linney, 1758 that there are two types of cytoplasmic factors: one initiates chromatin diminution, and the other inhibits it. This result was later confirmed by Moritz (1967) for *Parascarisequorum* (Nematoda) Goeze, 1782 and Geyer-Dushinskaya (1959) for the cecidomyiid *Wachtiella* Rübsaamen, 1916. Amma (1911) found in the cytoplasm of embryonic cells of 16 copepod species the presence of ectosomes, which are associated with the differentiation of germ line cells. Although chromatin diminution is not found in most of these species, Beermann (1977) suggests that mechanisms of diminution in the presomatic cells in *Cyclops* involves the synthesis and activation of a series of enzymes, which in copepods probably are set in action only during the long interphase preceeding the diminution division.

The goal of the present study is to determine whether a dose of radiation that blocks the action of the nuclear genome of *C.kolensis* embryos is capable of terminating the chromatin diminution processes. The results obtained will help us to understand when the factors of initiation of chromatin diminution processes appear or are activated: either in the cytoplasm of an unfertilized egg, or as a result of gene expression in the presomatic cells of *C.kolensis* embryos. To distinguish between these two alternative mechanisms, we chose the method of radiation inactivation of nuclei (Neyfakh 1961). This method is based on the fact that radiation doses of the order of 10–20 Gy destroy chromosomes, causing numerous aberrations, but allow the cytoplasm of embryonic cells to continue performing its functions. Such methods have not yet been used to study chromatin diminution.

## Materials and methods

Individuals of *C.kolensis* were collected from the small Andreevsky pond in Vorobievy Gory, Moscow, Russia (55°42'35.40"N, 37°34'6.61"E). This oligotrophic pond is located in a park area that prevents pollutants from entering into it, with the exception of pollutants that arise from atmospheric precipitation. Thus the impact of any significant exposure to chemicals that can affect the results of experiments on the individual’s body is practically eliminated. A necessary condition for the experiment was a method that allowed us to determine the stage of chromosomal fragmentation in developing *C.kolensis* embryos that could be assayed with a light microscope *in vivo*. The number of blastomeres in the embryos of *C.kolensis* corresponds to the number of spherical formations, which are sections of the cytoplasm with a nucleus that possess an optical density different from that of the surrounding yolk. Similarly, it is possible to reliably determine the stage of development of the embryo until the end of the 6^th^ cleavage division (Grishanin and Zagoskin 2019).

Irradiation of *C.kolensis* embryos was performed at the 4 cell stage with gamma (γ)-radiation. The amount of absorbed dose of ionizing radiation in the experiment varied from 5 to 200 Gray (Gy). The irradiation experiment was carried out in the Laboratory of Nuclear Problems of the Joint Institute for Nuclear Research at the Rokus-M facility. The absorbed dose rate was 1.37 G/min, delivered from the distance is 0.75 m. The irradiated embryos were examined for number of cells and any chromosomal irregularities 24 hours following irradiation. Fixation was performed with a mixture of ethanol and acetic acid in a ratio of 3:1 for one hour. The preparations were further stained with acetoorcein according to the method described earlier (Grishanin et al. 1996). Embryos were examined for presence of micronuclei at the interphase stage, indicative of chromosomal abnormalities. Statistical processing of the data was performed using the ‘*t*-test.’

## Results

**Figure 1.**
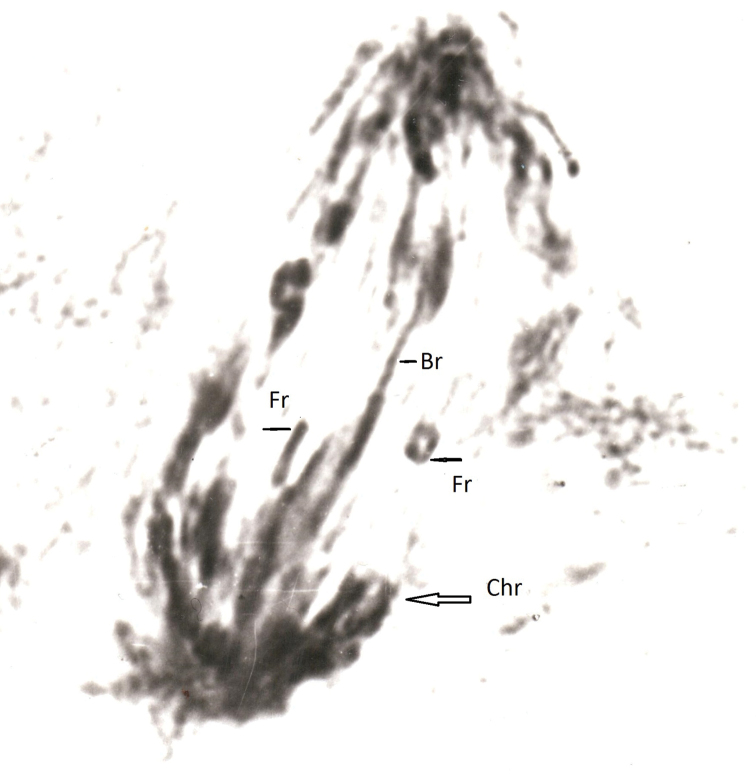
Anaphase of 3^rd^ cleavage division of *C.kolensis* embryo at 5 Gy (dose of ionizing radiation). Designations: Chr- chromosome, Br- bridge, Fr- fragment.

We initiated the experiment with a dose of 5 Gy, based on previous studies that showed a high level of chromosome aberrations in pre-diminution cleavage divisions under irradiation with this level of radiation (Grishanin and Akifiev 2005). We assumed that this dose could be considered as a starting point for studying the process of suppression of nuclear functions in *C.kolensis*. At 5 Gy dosage approximately half of the embryos reached the stage of 128–256 cells; the second half ceased their development at the stage of 32 cells (Table 1). Taking into account that chromatin diminution in *C.kolensis* occurs at the 4^th^ cleavage division, and visually observing granules of eliminated chromatin in cells at the 32-cell stage, we can state that chromatin diminution occurs in all embryos irradiated with a dose of 5 Gy. In the few anaphases that could be observed, we found numerous bridges, fragments, and fused chromosomes (Fig. 1). After irradiation of *C.kolensis* embryos with a dose of 20 Gy at the 4-cell stage, about 40% of embryos underwent only one-cell division, reaching the 8-cell stage of development, while the remainder of the embryos ceased their development at the 16-cell stage, having passed the chromatin diminution stage (Table 1).

**Table 1.** Cell stage and number of cells before and after exposure to gamma radiation in Gray (Gy) units.

Dose (Gy)	Initial cell stage of exposure	Cell stage after 24 hrs exposure	n	% of Embryos at cell stage after 24 hrs	Number of interphase figures	Average number of micronuclei ± SEM
5	1	128–256	86	52.4	n.a.	
		32	82	48.8	n.a.	
20	4	8	126	41.0	80	1.14 ± 0.35
		16	181	58.9	n.a.	
100	4	8	228	64.5	94	4.5 ± 0.6
		16	125	35.4	n.a.	
200	2	2	50	34.2	n.a.	
		8	60	41.0	n.a.	6.3 ± 0.8
		16	36(14)**	24.6 (9.5)	n.a.	
Control	1	238	>1000		202	

Number of embryos at a particular cell stage 24 hours after exposure is noted by n. Chromosomal abnormalities (micronuclei) are reported for cells at the interphase stage in which the granules did not obscure their presence; Standard deviation of the mean is denoted by SEM. n.a. – denotes data are not available. ** partial suppression of chromatin diminution.

**Figure 2.**
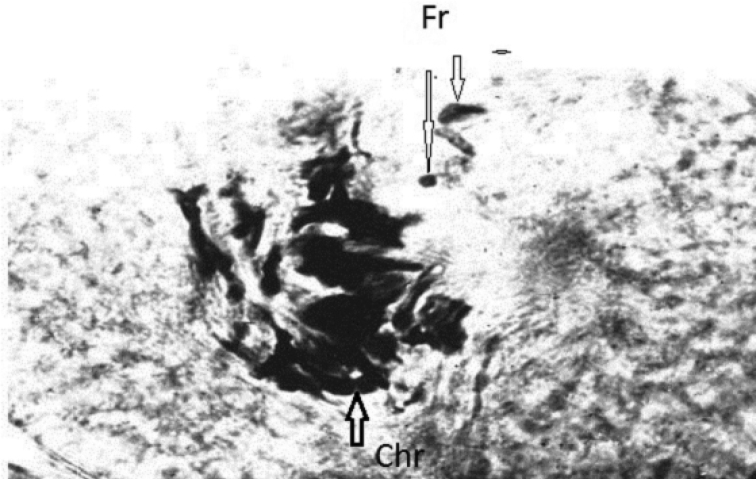
Metaphase of a *C.kolensis* embryo cell after irradiation at a dose of 100 Gy. Chr- chromosome, Fr- fragment.

*C.kolensis* embryos irradiated at the 4 cell stage with a dose of 100 Gy also comprised two similar groups: 65% of embryos ceased development at the 8-cell stage and, 35% of embryos ceased development at 16-cell stage, having passed the stage of chromatin diminution. Of those few anaphases that could be observed, we found numerous bridges, fragments and chromosomes adhering to each other (Fig. 2).

**Figure 3.**
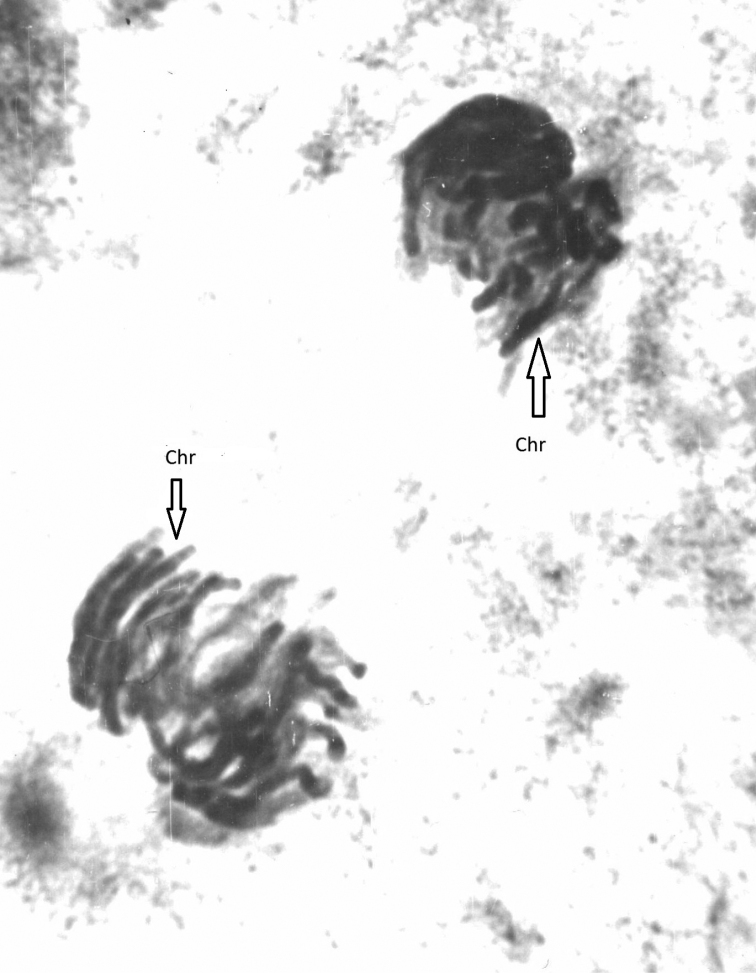
Anaphase of 3-d cleavage division of *C.kolensis* embryo in the control experiment. Chr- chromosome.

Irradiation with a dose of 200 Gy at the 2-cell stage apparently caused several disturbances in 34% of the embryos not only in the nucleus, but also in the cytoplasmic structures, as a result of which the development of the embryos stopped at the 2-cell stage. Nevertheless, more than half of the embryos reached the stage of the 8-cell embryo, and 25% of the embryos reached the stage of 16 cells, while in 14 out of 36 embryos chromatin diminution did not occur. We concluded that chromatin diminution did not occur in 14 embryos because we did not observe the usual pattern for embryos after chromatin diminution: an abundance of granules and sharp differences in size between cells of the somatic line before and after chromatin diminution. Damage to nuclear structures at a dose of 200 Gy was so significant that adhered chromosomes at the meta- and anaphase stages formed conglomerates and were not capable of segregation during cell division. No chromosomal aberrations were observed in the control embryos (Fig. 3). Numerous micronuclei were observed in 8-cell embryos at the interphase stage. With an increase in the dose from 20 to 200 Gy, an increase in the number of micronuclei per cell is observed (Table 1).

## Discussion

**Figure 4.**
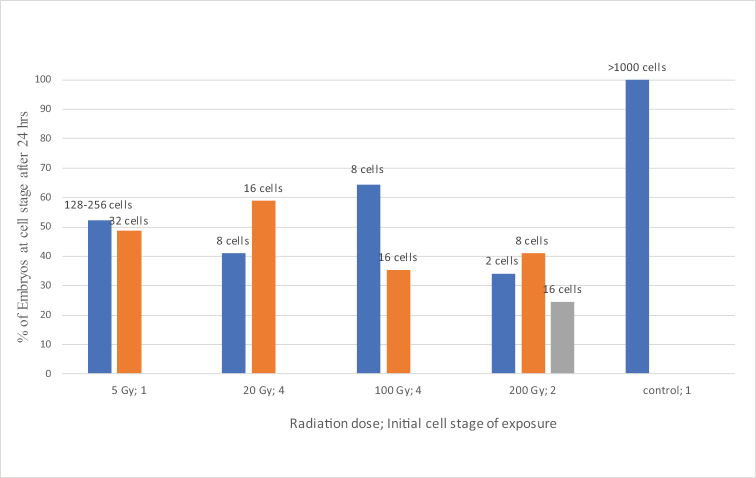
The percentage of *Cyclopskolensis* embryos that reached a particular cell stage of embryonic development in embryos exposed to different levels of radiation for 24 hours.

The results of experiments with embryos of *C.kolensis* at doses from 20 to 200 Gy have almost identical results: the development of embryos did not progress beyond the 16-cell stage (Fig. 4). The fact that some of the embryos ceased developing at the stage of 8-cell stage can be explained by damage to the cytoplasmic structures that ensure progression to 4^th^ cleavage division. In more than 30% of embryos irradiated with doses of 100 and 200 Gy, and in more than 50% of embryos irradiated with a dose of 20 Gy, development stopped at the stage of 16-cell stage and while the mechanism of chromatin diminution processes was realized. It can therefore be concluded that the development of embryos of *C.kolensis* with radiation inactivated nuclei up to the stage of 16 cells is determined by a development program and is regulated by the determinants in the egg cytoplasm. Having lost its potential, the embryo ceases its development. Numerous chromosomal aberrations may block the function of the nuclear genome of presomatic cells, and hence the morphogenetic function of nuclei. A similar trend in the dynamics of developmental processes during early embryogenesis was observed in the nematode *Ascarissuum* (Nematoda) Goeze, 1782. Irradiation of *A.suum* embryos at the 4-cell stage prevented them from developing past the 16-cell stage (Neyfakh 1961). Neyfakh’s intention was not to study the effect of γ-radiation on the chromatin diminution process in *A.suum. A.suum* undergoes chromatin diminution at the 3^rd^, 4^th^, and 5^th^ cleavage divisions (Niedermayer and Moritz 2000). Radiation blocked the function of the nuclear genome of *A.suum*, but did not suppress the chromatin diminution process.

We conclude that in the *C.kolensis* zygote, transcription factors and signaling molecules are expressed in precise patterns that determine the fate of somatic and germ line cells, and consequently the diminution processes in somatic line cells. High doses of gamma- radiation inactivate the hereditary information contained in the cell nuclei of *C.kolensis* embryos at doses up to 200 Gy, but do not affect the components of the cytoplasm that are important for patterning the chromatin diminution, which control the development up to the stage of 16-cells stage. But still, a dose of 200 Gy in some embryos was capable of disrupting the structure or dynamics of cytoplasmic factors that determine the possibility of chromatin diminution.

Thus, the results of the experiment confirm the assumptions of Boveri (1910), Geyer-Dushinskaya (1959) and Beermann (1977) in the cytoplasm of the embryos of the *Ascaris* Linnaeus, 1758, Cecidomyiidae and *Cyclops* Müller, 1785, species, in which the phenomenon of chromatin diminution is observed in early embryogenesis, there are factors that determine this process.

We did not attempt to compare the frequency of chromosomal aberrations at different doses of radiation using the methods of analysis of anaphases and counting micronuclei in the interphase stage. The relatively low frequency of chromosome aberrations, which we observed in this experiment, should be noted in comparison with earlier experiments on irradiation of *C.kolensis* embryos with the same dose but with a time interval of 60–180 min before embryo fixation (Grishanin and Akifiev 2005). Unfortunately, we could not observe the numbers of micronuclei present after irradiation of *C.kolensis* embryos at the 16- and 32-cell stages that were irradiated with doses of 20 and 100 Gy, since at these stages of development there were always a large number of granules of eliminated chromatin obscuring micronuclei. The tendency of an increase in the number of micronuclei per cell seems to occur naturally with an increase in the dose of γ-radiation.

The present study supports the following conclusions with regard to γ-radiation of *C.kolensis* embryos irradiated with a doses over 20Gy:

1. Development of C. kolensis embryos ceases at the 8–16 cell stage,
2. Initiation of the chromatin diminution program is not suppressed, except at the irradiation level of 200 Gy
